# Generation of Cholinergic and Dopaminergic Interneurons from Human Pluripotent Stem Cells as a Relevant Tool for In Vitro Modeling of Neurological Disorders Pathology and Therapy

**DOI:** 10.1155/2016/5838934

**Published:** 2016-12-25

**Authors:** Anna Ochalek, Karolina Szczesna, Paolo Petazzi, Julianna Kobolak, Andras Dinnyes

**Affiliations:** ^1^Molecular Animal Biotechnology Laboratory, Szent Istvan University, Pater K. Street 1, Godollo 2100, Hungary; ^2^BioTalentum Ltd., Aulich L. Street 26, Godollo 2100, Hungary; ^3^Bellvitge Biomedical Research Institute, Hospital Duran i Reynals, 3a Planta, Gran Via de l'Hospitalet 199, Hospitalet de Llobregat, 08908 Barcelona, Spain

## Abstract

The cellular and molecular bases of neurological diseases have been studied for decades; however, the underlying mechanisms are not yet fully elucidated. Compared with other disorders, diseases of the nervous system have been very difficult to study mainly due to the inaccessibility of the human brain and live neurons in vivo or in vitro and difficulties in examination of human postmortem brain tissue. Despite the availability of various genetically engineered animal models, these systems are still not adequate enough due to species variation and differences in genetic background. Human induced pluripotent stem cells (hiPSCs) reprogrammed from patient somatic cells possess the potential to differentiate into any cell type, including neural progenitor cells and postmitotic neurons; thus, they open a new area to in vitro modeling of neurological diseases and their potential treatment. Currently, many protocols for generation of various neuronal subtypes are being developed; however, most of them still require further optimization. Here, we highlight accomplishments made in the generation of dopaminergic and cholinergic neurons, the two subtypes most affected in Alzheimer's and Parkinson's diseases and indirectly affected in Huntington's disease. Furthermore, we discuss the potential role of hiPSC-derived neurons in the modeling and treatment of neurological diseases related to dopaminergic and cholinergic system dysfunction.

## 1. Introduction

The majority of underlying mechanisms related to human neurological dysfunction are not fully examined. Most of the current knowledge about neurodevelopmental and neurodegenerative disorders focuses on studies of postmortem nerve tissues, spinal cords, and brains or cancer tissue (e.g., neuroblastoma). Due to the limited access to neuronal samples from postmortem organs and the restricted possibilities for directly examining live human neurons, the current understanding of the cellular and molecular mechanism of these diseases is restricted. Additionally, studies with tissues from autopsies that often represent the end stage of the disease do not always reveal information on the course of disease. A significant contribution for elucidating the pathogenesis of various neurological abnormalities has been represented by transgenic animal models that can mimic human diseases [1]. Transgenic/knockout technologies provide a useful tool for investigation of disease mechanism. However, animal models do not fully recapitulate complex human disease phenotypes and very often are limited only for monogenic disorders.

Recent discoveries in pluripotent stem cell technology provide a new opportunity to overcome these limitations. Production of human pluripotent stem cells (hPSCs) from different somatic lineages can be seen as a novel tool that allows the development of treatments for human neurological disorders through disease modeling, drug screening, and regenerative medicine.

Human embryonic stem cells (hESCs) first established by Thomson et al. [2] and hiPSCs developed by Yamanaka's group [3], as the main types of hPSCs, both show unlimited self-renewal properties and the ability to differentiate into cells of all three germ layers [4].

Currently, many neurological diseases show dramatically increasing trends and, with the aging populations of developed countries, potential treatments are needed urgently. Nowadays, Alzheimer's and Parkinson's diseases are the most common progressive neurodegenerative disorders in aging populations. World Alzheimer Report estimated that 46.8 million people worldwide live with dementia and over 9.9 million new cases are detected each year. By 2050, the total number of people with dementia will increase to 135 million. According to available statistics published by European Parkinson's Disease Association, around 6.3 million people are affected with Parkinson's disease worldwide. Generation of defined neural subtypes from hiPSCs to replace affected neurons in the brain may be an effective method in central nervous system (CNS) disease treatment. Furthermore, proper in vitro human cellular pathomechanisms model would be relevant. So far many different protocols were used for hiPSCs differentiation that consequently led to generation of broad numbers of neuronal subtypes: dopaminergic, cholinergic, glutamatergic, GABAergic, motor neurons, peripheral sensory neurons, and medium spiny neurons of the striatum [5]. Importantly, in Alzheimer's and Parkinson's diseases, dopaminergic and cholinergic neurons are the most frequently affected groups of neurons.

Generation of functional dopaminergic neurons from hiPSCs described in various protocols is relatively robust and reproducible, while cholinergic neurons production still requires optimization and increase efficiency. Herein, we will focus on recent accomplishments in generation of dopaminergic and cholinergic neurons and their potential use in the development of novel therapies.

## 2. Generation of Specific Neural Subtypes through Pluripotent Stem Cells Differentiation

Neuronal formation and patterning are critical for the proper wiring of the brain and they occur mostly during embryonic neurogenesis. Neuronal progenitor cells located in the neuroectodermal layer of the embryo are induced by various signaling factors to differentiate into neurons and glia. Based on the progenitor location in the developing brain, different neuronal and glia cells are produced. For instance, progenitor cells located in the ventral neural tube generate motor neurons and oligodendrocytes, while interneurons and astrocytes are produced from dorsal progenitor cells [6]. In addition to the position along the neuraxis, the fate of single neurons depends on many factors including epigenetic profile and patterning factors. Unspecified progenitor cells within the neuroectoderm can differentiate into various neural subtypes by modulating signaling pathways in which are involved bone morphogenic protein (BMP), Wingless-Type MMTV Integration Site Family (WNT) proteins, fibroblast growth factor (FGF), retinoic acid (RA), and other signaling molecules.

Numerous laboratories have established in vitro differentiation protocols to generate neurons. Initially, the methods were based on hESCs, although their use has been a source of ethical, legal, and social controversy, because of their derivation from early embryos. In 2007, Yamanaka's group has generated human iPSCs via genetic reprogramming of somatic fibroblast cells through retroviral transduction of four human transcription factors: POU domain, class 5, transcription factor 1 (*POU5F1*), better known from its former name octamer-binding transcription factor 4, and therefore often abbreviated as* OCT4*, sex determining region Y-box 2 (*SOX2*), Kruppel-like factor 4 (*KLF4*), and* c-MYC* [3]. Since this discovery, hiPSCs have been considered an important source for the generation of neurons and many other types of cells. iPSCs are similar to ESCs in many aspects such as the expression of pluripotency-related genes/proteins, embryoid body formation, teratoma formation, and capability of differentiating into all three germ layers. However, the full extent of iPSCs relation to ESCs is still being assessed.

Theoretically, iPSCs and ESCs both share the property of pluripotency that can be equally used in human disorders modeling. Despite many similarities between iPSCs and ESCs, there are several differences that have significant influence on new cell generation. For instance, the analysis of gene expression profiles revealed changes between cell lines based on their origin. The “epigenetic memory” of the original somatic cells may cause some specific aberrations which impede iPSC differentiation potential [7]. As a result of prolonged in vitro propagation and the environmental culture conditions, the genome integrity of iPSCs can be impaired. Consequently, genetic aberrations may decrease the reprogramming efficiency.

**Figure 1:**
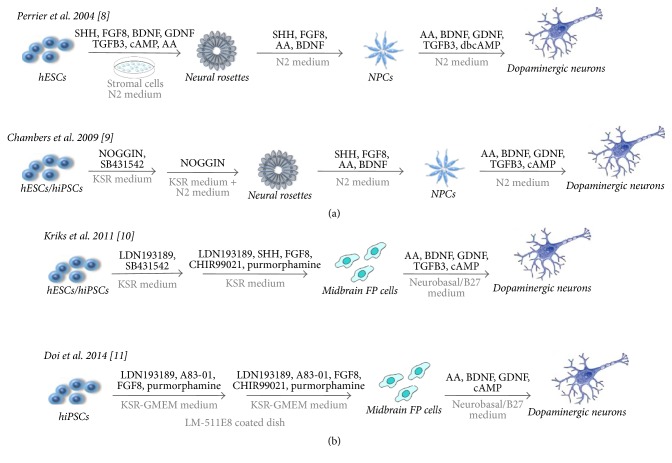
Comparison of different protocols for deriving of midbrain dopaminergic neurons from hESCs and hiPSCs. (a) Methods based on mechanical neural rosettes selection. Neural differentiation was induced by coculture of hPSCs on stromal cells MS5 or S2 [8] and dual inhibition of SMAD signaling pathway (NOGGIN + TGFB inhibitor: SB431542) in the presence of knockout serum replacement (KSR) and N2 medium [9]. Rosettes structures were harvested mechanically and gently replated in the presence of growth factors. In the final step, newly generated neural progenitor cells were differentiated into DA neurons in the absence of SHH and FGF8. (b) Methods based on the floor plate (FP) induction. Dual SMAD inhibition (BMP inhibitor: LDN193189 + TGFB inhibitor: SB431542) and activation of WNT signaling by SHH and GSK3B inhibitor (GSK3Bi), CHIR99021, were used for midbrain FP cell generation from hPSCs [10]. Purmorphamine treatment was applied for FP cell patterning. hPSCs induced with LDN193189 and A83-01 (inhibitor of TGFB type I receptor ALK5) were cultured in media supplemented with purmorphamine and FGF8 to induce floor plate cells. FP cells under stimulation with growth factors generated DA neurons; recombinant E8 fragments of human laminin 511 (LM511-E8) supported the neural differentiation and cell survival [11]. Final concentration of growth factors, supplements, and inhibitors may be different in the specified protocols.

**Figure 2:**
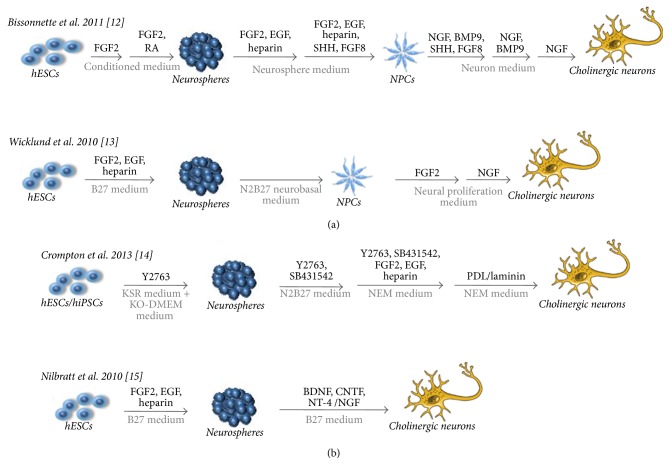
Comparison of different protocols for deriving of basal forebrain cholinergic neurons from hESCs and hiPSCs. (a) Generation of BFCNs from neurospheres through neural progenitor cell stage. Predominantly pure population of basal forebrain cholinergic neurons (BFCNs) was derived from hPSCs by using diffusible ligands presented in the forebrain during a development [12]. Pretreatment with SHH and FGF8 was used to differentiate hPSCs towards a forebrain progenitor fate [12]. Treatment with NGF promoted the differentiation into functionally mature BFCNs [12, 13]. (b) Direct generation of BFCNs from cells growing in neurospheres. Neural development was induced in neurosphere-based nonadherent differentiation in presence of mitogens and ROCK inhibitor Y27632 [14]. Neurospheres derived from hPSCs exposed to neurotrophins, BDNF, CNTF, NT-4, and NGF, increased neuronal differentiation and cholinergic phenotype specification [15]. Composition of the basic medium can be different within the above protocols.

Despite these not fully examined issues, several alternative differentiation protocols to obtain dopaminergic (Figure 1) and cholinergic neurons from hPSCs (Figure 2) have been developed. Human PSCs have potential to differentiate into functionally specialized cell types through the mechanism mimicking the in vivo development. Many factors such as media composition, concentration of signaling factors, plating cell density, and timing of differentiation and maturation process and physical parameters of culture system have significant influence on the type and efficiency of generated cells, such as neurons. In the next sections, we compare the different techniques generating midbrain dopaminergic neurons and basal forebrain cholinergic neurons from hPSCs. Figures 1 and 2 provide a comprehensive picture about the used methods, chemical compounds, and laboratories which published the methods. We also detailed whether the method was tested with different PSC cell sources (hESC or hiPSC).

### 2.1. Generation of Dopaminergic Neurons

Successful conversion of PSCs to dopaminergic (DA) neurons depends on the patterning and signaling factors that induce gene expression typical for endogenously developing DA neurons.

Neural stem cells (NSCs) are produced from iPSCs/ESCs that undergo induction in coculture with stromal feeder cells such as PA6 and MS5 [8, 16] or in feeder independent system. Neuroepithelial structure formed by NSCs is composed of rosettes that express neural markers like nestin (NES), paired box 6 (PAX6), sex determining region Y-box 1 (SOX1), and neural cell adhesion molecule (NCAM). Culture of NSCs in chemically defined neural induction medium results in differentiation of progenitor cells into different neuronal subtypes.

One of the first experiments performed on NSCs revealed that treatment of hESC-derived neural precursors with fibroblast growth factor 8 (FGF8) and sonic hedgehog (SHH) significantly increases the number of neurons expressing tyrosine hydroxylase (TH) and consequently the percentage of mature DA neurons [17]. The presence of glial cell line derived neurotrophic factor (GDNF) in culture medium additionally increases DA neuron differentiation [18].

Although FGF8 and SHH determine DA phenotype, the regional identity of the DA neurons (forebrain or midbrain phenotypes) can be specified under a chemical-defined media composition. Recently, two groups published protocols for generation of two different types of DA neurons; however, these studies used mouse ESCs (mESCs). In the first one, progenitor cells were differentiated into midbrain DA neurons in the presence of FGF8, SHH, and ascorbic acid (AA) and their maturation was confirmed by dopamine release in the medium [19]. In the second study, stromal cells were used to induce neuronal differentiation of mESCs in serum-free conditions without any patterning factors. These stromal derived cells induced significant increase of activity that is reflected in the number of TH-positive neurons producing dopamine. DA neurons after transplantation into the mouse striatum remain TH positive, thus indicating being more forebrain-like than midbrain-like dopaminergic neurons [20]. Additional studies showed that SOX1 positive neuroepithelial cells derived from hESCs and being exposed to FGF8 and SHH generate bipolar forebrain DA neurons that expressed TH enzyme. Treatment of neuroepithelial cells in precursor stage before* SOX1* expression leads to formation of midbrain DA neurons with large cell bodies and a specific midbrain marker, engrailed 1 (EN1) [21]. In most of the studies, the phenotype of DA neurons is confirmed by a wide panel of morphological and functional characteristics as follows: TH expression, KCl-evoked DA release, and presence of depolarization–induced and tetrodotoxin-sensitive action potential [8]. However, the regional identity of DA neurons is not well examined due to the lack of reliable antibodies (e.g., restricted detection of dopamine transporter (DAT), mature marker in cultured human DA neurons) and different time points for release of transcription factors and DA neuronal marker expression. In mESCs,* En1* transcription factor expression is overlapped with TH being downregulated in postmitotic TH neurons. Consequently, only low percentage of TH neurons coexpressed* En1* during neuronal differentiation [22]. In addition, the maturation and functionality of DA neurons are tested in 6-hydroxydopamine- (6-OHDA-) lesioned rats. Transplantation of DA neurons derived from hPSCs improves the behavioral deficits [11]. Analysis of grafts suggests that the majority of DA neurons are generated from grafted neuronal cells and some of them can be integrated into the striatal circuitry. Dopamine release from injected neural cells triggers functional improvements in rats [22].

Fork head box protein A2 (FOXA2) is another player with important influence in the early development and later maintenance of midbrain DA neurons. Midbrain dopaminergic neurons with a stable phenotype defined by expression of FOXA2, TH, and *β*-tubulin were generated from hESCs treated with low dose of retinoic acid and high activity form of SHH. Early exposure to WNT1 and FGF8a rather than FGF8b was required for efficient differentiation of the neural progenitor cells from the floor plate (FP) into the midbrain DA neurons that express* FOXA2* [23].

In 2009, Chambers et al. described the rapid and efficient conversion of hESCs and hiPSCs to neurons by the synergistic action of two inhibitors of SMAD pathway, NOGGIN, and SB431542 [9]. Single inhibition of SMAD pathway by SB431542 is associated with a dramatic decrease in the expression of* NANOG* and increase in caudal type homeobox 2 (*CDX2*) that consequently result in loss of pluripotency and differentiation through the trophoblast lineage. NOGGIN downregulates* CDX2* expression and represses BMP release that drive trophoblast destiny. Only dual SMAD inhibition significantly improves the neural induction of hESCs and hiPSCs under adherent culture conditions. Exposure of cells generated via this method to SHH at day 5 and to FGF8 at day 9 of terminal differentiation and further culture until day 19 in medium supplemented with AA, brain derived neurotrophic factor (BDNF), GDNF, transforming growth factor beta 3 (TGFB3), and cyclic adenosine 3′,5′-monophosphate (cAMP) leads to production of Tubulin, Beta 3 Class III (TUBB3) positive neurons coexpressing TH [9].

Another method of midbrain DA neurons generation is based on a FP strategy. FP is formed by a group of cells with neurogenic potential located at the ventral midline of the developing neural tube. Cells in this area secrete diffusible molecules such as SHH and Netrin 1 (NTN1) that are involved in neural tube patterning and guidance of extension of commissural axons [24]. The neurogenic potential of FP cells is established by expression of transcription factors, such as* EN1*, orthodenticle homeobox 2 (*OTX2*),* FOXA2* and LIM homeobox transcription factor 1 alpha (*LMX1A*), which are involved in two regulatory feedback loops (SHH-FOXA2 and WNT1-LMX1A) [25]. Differentiation of PSCs to FP cells and then activation of neurogenesis are an alternative way of DA neurons generation. Human ESCs exposed to high concentration of SHH change the morphology and properties towards FP cells [26]. Alternatively, SHH together with WNT can be utilized to drive hPSCs to midbrain FP precursors. Generated in this way, midbrain DA neurons can be maintained several months in vitro, and, after transplantation in 6-OHDA-lesioned mice and rats, they demonstrate complete restoration of movement activity [10]. WNT1 expressed in the ventral midbrain play a critical role in activating the neurogenesis. In consequence, regulation of WNT1 and SHH in neural progenitor cells can lead to generation of midbrain DA neurons via the alternative route.

### 2.2. Generation of Cholinergic Neurons

Basal forebrain cholinergic neurons (BFCNs) are the major source of cortical cholinergic input that is necessary for memory and learning. Previous studies in animals reveal important role of BFCNs in hippocampal neurogenesis [27] and functional synaptic plasticity in developing cortex [28]. The largest and the best characterized group of forebrain cholinergic neurons is placed in the median ganglionic eminence (MGE). BFCNs are primarily affected in Alzheimer's disease. For this reason, in vitro generation of cholinergic neurons from human PSC is a crucial step for disease modeling and novel cell replacement therapy for Alzheimer's patients. However, the number of BFCNs differentiated from hPSCs is still very limited and the regional identity of generated neurons is not well characterized.

One of the first methods for the derivation of BFCNs from hESCs was based on the stimulation using diffusible ligands present in the MGE at developmentally relevant time periods [12]. The crucial step in the above experiment was differentiation of hESC-derived neural progenitor cells towards a forebrain progenitor fate by pretreatment with SHH and FGF8. Neural progenitors that are generated in this way and treated transiently with bone morphogenic protein 9 (BMP9) showed a significant increase in the expression of BFCN markers such as choline acetyltransferase (ChAT), acetylcholinesterase (AChE), and nerve growth factor receptors (NGFR): TrkA and neurotrophin p75. Markers for other populations of cholinergic neurons were not detected. Direct treatment of hESCs with BMP9 without SHH/FGF8 pretreatment resulted in the absence of cholinergic neurons. MGE during development expresses some transcription factors including LIM homeobox 8 (*LHX8*) transcription factor [29] and gastrulation brain homeobox 1 transcription factor (*GBX1*) that promote BFCN differentiation [30] and are upregulated by BMP9. Transiently overexpression of* LHX8* and* GBX1* in SHH/FGF8 pretreated neural progenitors leads to appearance of a highly purified population of BFCNs with long projecting axons and coexpression of ChAT and p75 [12]. These neurons produce acetylcholine in vitro and form cholinergic synapses which were electrophysiologically functional after engraftment into murine hippocampal slice cultures.

Another method of active ChAT-positive cholinergic neurons generation focused on neurotrophic factors. The neurotrophins are growth factors that act by stimulating Trk and p75 receptors, which in turn are responsible for axonal and dendritic growth, neurotransmitter regulation, and synaptic plasticity [31]. hESCs-derived neuronal cells after stimulation with neurotrophins, BDNF, neurotrophin 3 (NTF3), ciliary neurotrophic factor (CNTF), and nerve growth factor (NGF), significantly increase the proportion of ChAT-positive neurons [15]. However, stimulation of neural progenitors with BDNF and NGF upregulates* LHX8* expression in MGE areas of the embryonic forebrain, whereas CNTF induce LIM homeobox 6 transcription factor (*LHX6*) expression in other subdomains of the MGE. Additional expression of NK2 homeobox 1 (*NKX2-1*) and ISL LIM homeobox 1 (*ISL1*) is associated with the development of forebrain cholinergic neurons and coexpression of p75 receptor demonstrated the presence of BFCNs. Furthermore, these differentiated neuronal cells produce *α*3, *α*4, and *α*7 subunits of nicotinic acetylcholine receptor and muscarinic acetylcholine receptor subtypes, M1, M2, and M3, that are displaying an important role in hESCs-derived cholinergic neurons [15]. All the above-mentioned methods used specific extrinsic factors to produce cholinergic neurons in two-dimensional cultures. However, the latest study has reported a novel embryoid body based differentiation system for efficient induction of BFCNs [14]. Cultures of embryoid bodies in 3-dimensional systems without any additional factors stimulate intrinsic SHH signaling which results in expression of* NKX2-1* and* LHX8* [32]. Upon terminal differentiation, the basal forebrain specific NSCs generate electrically active cholinergic neurons that express TUBB3, ChAT, ISL1, and p75 and after transplantation are able to integrate into the adult rat brain [14]. In the newest study published by Hu et al., hPSCs were converted to NKX2-1 positive MGE cells by using high concentration of SHH or combination of SHH and purmorphamine. To increase the efficiency of BFCNs generation, MGE progenitor cells were cocultured with hPSC-derived astrocytes in the presence of NGF [33]. In the above method, around 40% of total cell population has expressed cholinergic markers that offers a potent approach to produce BFCNs from pluripotent cells.

## 3. iPSCs Providing New Tools for Developing Treatments for Cholinergic and Dopaminergic System Dysfunctions

Current understanding of the etiology of neurological disorders is greatly expanded. However, the mechanisms of most of these diseases remain still unclear. Furthermore, no effective treatments or disease-modifying therapy are available. Thus, patient-derived human iPSCs are a good tool for generating physiologically relevant in vitro human disease models. Stem cells technology can be applied in disease pathomechanism investigations, identification of potential drug targets, drug screening platforms, and cell transplantation.

Newly generated iPSCs from patient-derived tissues are successfully applied in modeling of human neurodegenerative diseases (e.g., Alzheimer's disease, Parkinson's disease, spinal muscular atrophy (SMA), amyotrophic lateral sclerosis (ALS), and Huntington's disease) and neurodevelopmental disorders (e.g., familial dysautonomia, Rett syndrome, autism spectrum disorder (ASD), and Down syndrome) (Table 1). In recent years, the dopaminergic and cholinergic systems have been a main focus of research in neurological pathogenesis. Efficient generation of functional dopaminergic and cholinergic neurons affected mostly in Parkinson's disease and Alzheimer's/Huntington's disease patients, respectively, can provide a crucial tool for effective treatment of the indicated dysfunctions.

### 3.1. Alzheimer's Disease and iPSCs

Alzheimer's disease (AD) is an age-related neurodegenerative disorder and the most common type of dementia in humans, affecting one in eight adults over 65 [34]. Two main pathological hallmarks of AD are extracellular plaques of aggregated amyloid beta (A*β*) protein and intracellular neurofibrillary tangles (NFTs) composed of aggregated tau, a microtubule binding protein. These AD features have different deleterious effect on neurons. A*β* can disrupt synaptic plasticity including long-term potentiation (LTP), while NFTs may compromise intracellular transport and, together with A*β*, induce mechanisms responsible for synaptic loss and neuronal death.

Neurodegeneration in AD evolves sequentially through certain brain regions and selected subpopulations of vulnerable neurons. Among the general cortical impairment, BFCNs are one most affected cell type in AD. Decreased level of acetylcholine (ACh) released by cholinergic nerve terminals is associated with loss of cholinergic neurotransmission and significant deterioration of cognitive functions in AD patients. Reduced choline uptake, ACh release, and presynaptic cholinergic deficiency correlate with accumulation of A*β* protein and intracellular NFTs. Furthermore, analysis of AD brain revealed reduced number of nicotinic and muscarinic M2 ACh receptors on presynaptic cholinergic neurons, whereas the number of M1 and M3 receptors on the postsynaptic terminals has remained unaffected. Some studies have shown that reduced cholinergic activity may stimulate higher tau hyperphosphorylation through increased activity of protein kinase C (PKC) [35]. As a consequence of disturbed neurotransmission balance, tau phosphorylation and A*β* protein accumulation are increased, and these lead to enhanced neurodegeneration. BFCNs degeneration contributes to attention deficits, increased spatial memory decline, and further impairment in the coding of new episodic memories [36].

Centrally acting drugs that inhibit cholinesterase such as tacrine, donepezil, rivastigmine, and galantamine have been shown to provide modest symptomatic benefit in individuals with AD [37]. AChE inhibitors together with muscarinic receptor agonists are able to restore cholinergic balance and decrease A*β* deposition [38].

Recently, some groups have developed an AD disease model using iPSCs. Neurons derived from iPSCs generated from familial AD (fAD) patients carrying mutations in genes encoding amyloid precursor protein (APP), presenilin 1 (PSEN1), and presenilin 2 (PSEN2) provide an innovative tool to elucidate AD etiology and develop efficient therapeutics.

The sequential proteolytic cleavages of APP by *β*-secretases and *γ*-secretases result in the generation of A*β*. While *γ*-secretases cleave C-terminal fragments of APP (APP-CTF) which leads to production of multiple length variants of A*β*, longer variants of A*β* (A*β*42, A*β*43) are more prone to aggregation than shorter ones (A*β*38, A*β*40) and they are considered more pathogenic [39].

Kondo et al. generated an AD iPSCs model from patients with E693 deletion in APP gene. Neurons with this mutation accumulated intracellular A*β* oligomers that led to endoplasmic reticulum (ER) and oxidative stress. Furthermore, the accumulated A*β* oligomers were not proteolytically resistant, and the treatment of AD neural cells with docosahexaenoic acid (DHA) alleviated the stress responses [40]. These results suggest that DHA may be an effective drug for a subset of patients, making iPSC technology a useful tool for validation and identification of potential drugs. Muratore et al. described iPSCs from fAD with mutation in* APP* (V717I). This mutation results in higher APP expression, elevated A*β*42, A*β*38 production, and increase in levels of total and phosphorylated Tau in neurons [41]. In another study, iPSCs derived neurons from fAD patients with a duplicated APP gene showed increase of secreted A*β*1–40, relative level of phospho-Tau, and active glycogen synthase kinase 3 beta (GSK3B). Treatment of the neurons with *β*-secretase inhibitors significantly reduced the relative level of phospho-Tau and active GSK3B [42]. fAD-derived iPSCs with PSEN1 (A246E) and PSEN2 (N141I) mutations were also established. In both cases, neuronal cells had increased A*β*42 secretion and A*β*42/40 ratio [43]. The treatment with *γ*-secretase inhibitors reduced A*β*40 and A*β*42 production in mutant neurons. The above-presented findings on iPSCs derived from patients are a good method of recapitulation of AD phenotype in vitro and can be used for drug discovery and improve disease modeling.

In recent time, few groups successfully generated iPSCs from sporadic AD (sAD) patients that represent more than 95% of all AD cases. sAD iPSCs showed similar phenotype to fAD iPSCs including increase of secreted A*β*, high level of ER and oxidative stress, and accumulation of enlarged RAB5-positive early endosomes [42]. However, there is still limited information about the clinical onset and course of sAD. Newly developed models, mostly based on 2-dimensional (2D) culture system, may not reflect affected neurons in the brain. Due to the limited studies, it is difficult to predict whether neurons derived from sAD iPSCs show pathological phenotype and are able to reveal molecular basis of disease development. To evaluate a real value of sAD derived iPSCs, a long-term culture in 3-dimensional (3D) system may be required. Furthermore, integration of microglia in neuronal culture together with environmental cues mimicking sAD risk factors might significantly improve in vitro-based experimental sAD models.

According to the recent data, animal and nonneuronal cellular models used in pharmaceutical compound validation cannot sufficiently model the drug responses of human neurons. Therefore, there is an increasing trend to test potential drugs for the treatment of Alzheimer's disease using patient-derived iPSC based systems.

Based on the fact that mutation in APP or in the *γ*-secretases components, PSEN1 and PSEN2, lead to increased A*β*42/A*β*40 ratio and total A*β* level, several drugs such as *β*-secretase and *γ*-secretase inhibitors have been developed [44, 45]. Nonsteroidal anti-inflammatory drugs (NSAIDs) were identified as *γ*-secretase modulators (GSMs) that lower A*β*42 production by targeting *γ*-secretase or APP [46]. The studies performed on iPSC-derived neurons from AD patients have shown that indometacin, ibuprofen, diclofenac, and flurbiprofen significantly reduced the A*β*42/A*β*40 ratio [46]. Treatment with *γ*-secretase inhibitor, N-[N-(3,5-difluorophenacetyl)-1-alanyl]-S-phenylglycine t-butyl ester (DAPT), or *γ*-secretase activating protein (GSAP) inhibitor, imatinib, resulted in strong decrease of A*β*40 and A*β*42 level. Inhibition of A*β*40 secretion by SC-560 led to increased A*β*42/A*β*40 ratio. Other tested molecules including aspirin, naproxen, and Rho-associated coiled-coil forming protein serine/threonine kinase (ROCK) inhibitor Y-27632 had no significant effect on the neurons [46].

### 3.2. Parkinson's Disease and iPSCs

Parkinson's disease (PD) is the second most common progressive neurodegenerative disorder. Patients with PD manifest a wide range of symptoms, encompassing slowness of movement, rigidity, a low-frequency rest tremor, and difficulty with balance. These crucial motor impairments of PD are due to the degeneration of dopamine containing neurons in the substantia nigra pars compacta (SNC) with an accompanying loss of dopamine and its metabolites in the striatum [47].

The majority of PD cases are sporadic; only 10–20% patients present familial monogenic form of disease. Mutations in genes, synuclein alpha (*SNCA*), leucine-rich repeat kinase 2 (*LRRK2*), Parkin RBR E3 Ubiquitin Protein Ligase (*PARK2*), PTEN-induced putative kinase 1 (*PINK1*), ubiquitin carboxyl-terminal esterase L1 (*UCHL1*), and beta-glucocerebrosidase (*GBA*), lead to pathogenic changes in the brain [48].

At the pathological level, PD is characterized by the cytoplasmic accumulation of aggregated proteins with a halo of radiating fibrils and a less defined core known as Lewy body. Another PD-specific feature is the increase of oxidative stress, which is caused by glutathione depletion, iron deposition, increased markers of lipid peroxidation, oxidative DNA damage and protein oxidation, and decreased expression and activity of mitochondrial complex 1 in the SNC [49].

Several groups revealed dysfunction of basal forebrain cholinergic system in PD patients that was confirmed by the presence of Lewy body in neurons of the nucleus basalis of Meynert, the main source of cholinergic neurons in the brain [50]. Additional study has indicated a loss of presynaptic cholinergic markers in cortex, decrease of muscarinic and nicotinic receptor binding sites, and reduced ChAT activity in the neocortex, hippocampus, and substantia nigra. Impaired distribution of pre- and postsynaptic cholinergic receptors can correlate with PD attenuation.

Recent data have shown that the loss of DA neurons in PD corresponds with alterations of M4 receptor in cholinergic neurons. In consequence, ACh level in the striatum is increased and that contributes to the development of the motor signs like tremors and dyskinesia in PD patients [35].

Unlike most other neurodegenerative disorders, there is an effective temporary symptomatic treatment for PD consisting of dopamine replacement with levodopa or dopamine agonists [51]. On the other hand, since the neurodegeneration in PD is progressive and there is no efficient regenerative therapy, patients eventually become quite disabled. Therefore, the inhibition of postsynaptic muscarinic receptor resulting in ACh secretion decrease and upregulation of presynaptic nicotinic receptors secreting dopamine can provide a new therapeutic strategy in PD treatment.

The iPSC technologies provide an opportunity to study PD phenotypes and mechanisms in familial and sporadic cases and to identify potential drugs.

One of the first models of familial PD was iPSCs generated from a patient with triplication of the SNCA gene, which encoded a synaptic vesicle-associated protein in Lewy bodies. DA neurons derived from this patient exhibit increased* SNCA* expression and susceptibility to oxidative stress [52]. Other researchers generated iPSCs from patients with the most common SNCA mutation, A53T. Compared with controls, these cells produced more nitric oxide and 3-nitrotyrosine (3-NT) and showed accumulation of ER-associated degradation substrates [53]. DA neurons differentiated from iPSCs derived from patients with G2019S mutation in* LRRK2* gene demonstrated increased expression of key oxidative stress response genes:* HSPB1*,* NOX1*,* MAOB*, and upregulation of* SNCA*. The mutant neurons were also more sensitive to stress agents: hydrogen peroxide, the proteasome inhibitor MG132, and 6-OHDA [54]. Another study showed that DA neurons developed from iPSCs of either sporadic PD or LRRK2 PD showed similar phenotypes including higher level of SNCA, the accumulation of SNCA, and reduced numbers of neurites [55]. If these results will be reproducible, they may give us a possibility to model also idiopathic PD cases in the future. Despite the unknown causes of this disorder, a small proportion of idiopathic PD cases can be attributed to known genetic factors. Although generation of iPSCs from idiopathic PD patients was established, it is still not confirmed whether PSC-derived DA neurons demonstrate phenotypes that are evidently detectable and related to PD in vitro [56]. Due to the unknown mechanisms leading to sporadic PD, that may result from a combination of genetic and environmental factors, the use of isogenic control for comparative studies is not possible. However, an increased number of cell lines generated from patients could make iPSC models more reliable in the context of complex diseases such as sporadic PD.

The main DA neurons were also generated from iPSCs carrying mutation in* PINK1*. This gene encodes a mitochondrial kinase which protects cells against mitochondrial stress and control degradation of mitochondria. DA neurons with this defect upon treatment with the mitochondrial stress inducer increase level of peroxisome proliferator-activated receptor gamma, coactivator 1 alpha (PPARGC1A) regulator of mitochondrial biogenesis, and the number of mitochondria [57]. The above results show that not only genetic background of neurons but also their exposure to the relevant stressors may be necessary for proper modeling of disease phenotype using iPSCs. DA neurons carrying the mutation in PARK2 gene increase the transcription of monoamine oxidases and significantly block DA uptake that leads to higher DA release. Insertion of the correct form of the mutant gene rescues the phenotype. It suggests that PARK2 controls dopamine neurotransmission and suppresses dopamine oxidation [58]. These results provide a physiologically relevant platform to screen the novel targets of disease-modifying therapies in PD.

Recently, it was shown that iPSCs can be successfully transplanted into PD animal models. iPSCs efficiently stimulated into NPCs and injected into the brain give rise to neuronal and glia cells that are functionally integrated with the neural tissues and have mature neuronal activity. Midbrain DA neurons differentiated from hiPSCs upon transplantation into the adult brain were able to improve the behavioral phenotype of rats affected by PD [59]. Another study showed that DA neurons generated from PD iPSCs and transplanted into the striatum of 6-OHDA-lesioned rats developed axons projections [60]. Rhee et al. demonstrated that transplantation of DA neurons derived from protein-based hiPSCs into rats with striatal lesions can rescue motor deficits [61]. In 2005, Kishi et al. have demonstrated a possible role of estrogen in the transplantation of NSCs for Parkinson's disease. 17*β*-estradiol, estrogen hormone, increased the number of TH-positive DA neurons in vitro. The above effect was abrogated by an estrogen receptor antagonist, ICI182780, that confirmed a role of ERs in differentiation of DA neurons [62]. Due to the presence of ERs in NSCs and DA neurons, increase in TH-positive neurons was associated with the supporting effect of estrogen on neural differentiation of iPSCs and DA neuron maturation [62].

### 3.3. Huntington's Disease and iPSCs

Huntington's disease (HD) is an inherited progressive disorder that affects around 1 in 10,000 people. The specific features of HD comprise dystonia, motor incoordination, and cognitive and emotional impairments [83]. Cause of the disorder is an expanded CAG triplet coding for polyglutamine in the HD gene product, called the huntingtin (HTT) protein. The normal function of this protein is poorly understood with some evidences of an involvement with cytoskeletal function.

At the pathological level, HD is characterized by selective neuronal vulnerability. The first area affected by HD is the caudate-putamen that is part of the corpus striatum region. Within this brain region, medium spiny GABA neurons are severely impaired, with up to 95% loss in later stages of disease (reviewed in [84]). Moreover, there are intranuclear inclusion bodies and perinuclear and neuritic aggregates of HTT in HD neurons. Early genetic studies on HD revealed that the length of the triplet repeat expansion of CAG is positively correlated with the severity of the disease. Glutamate toxicity, oxidative stress, and autophagy inhibition are stressors that stimulate CAG expansion and result in higher cell toxicity [5].

Some studies in human and mouse models indicated that also cholinergic system is affected in HD patients. The cholinergic neuron abnormalities were demonstrated by the observations that ChAT activity is reduced in the striatal tissue of the brain and ACh release is significantly decreased [85]. It was confirmed that vesicular acetylcholine transporter (VAChT) and ChAT genes are regulated by the repressor element-1 silencing transcription factor/neuron restrictive silencer factor (REST/NRSF) [86]. Interaction of REST/NRSF with mutant HTT protein leads to repression of few neuronal specific genes such as BDNF and can cause a decrease of cholinergic marker expression. During the studies performed by Smith et al., decreased level of ChAT in striatum of HD mice correlated with the amount of HTT aggregates in cholinergic neurons. These results suggest that the defect observed in cholinergic neurons depends on the mechanism controlled by mutated HTT [85].

Recently, AChE inhibitors such as tacrine were proposed to be potential drugs for HD treatments due to their ability to restore the cholinergic system. Choline, physostigmine, donepezil, and other potential drugs in HD treatment are being tested now; however, their ability to restore a memory and reduce the hyperkinesia seems to be very limited [35].

HD neural cells derived from iPSCs displayed disease associated phenotype including changes in metabolism, electrophysiology cells adhesion, and cell toxicity. Differentiated HD cells with expanded CAG repeats showed changes in gene expression, including cadherin family and TGFB pathways. Additionally, cells had abnormalities in oxygen consumption and were more vulnerable to cell stressors and BDNF withdrawal [69]. In other studies, HD-generated iPSCs presented impaired lysosomal activity, mitochondrial fragmentation, and alterations in transcription repressor activity [5]. Park et al. differentiated HD specific neural stem cells derived from patient iPSC into striatal neurons. In these neuronal cells, an enhanced caspase 3/7 activity was detected [70]. HD iPSCs after treatment with inhibitor of mitochondrial fission related protein: dynamin 1-like (DNM1L) increased cell viability [66]. During in vivo studies performed by Jeon and coworkers, HD-derived neuronal progenitor cells were transplanted into HD rat with excitotoxic striatal lesions. Behavioral recovery was observed, even though the injected cells have revealed HD phenotype [87]. Above studies suggest that mutation correction in iPSCs is required for effective treatment of HD.

Drug screening and test of novel therapies using HD iPSC-derived neuronal cells include application of stressors such as glutamate toxicity, oxidative stress, DNA damage, or growth factor withdrawal [88] to test molecules counteracting the effects. For instance, the tumor necrosis factor-alpha (TNF*α*) inhibitor XPro-1595 decreased cytokine induced apoptosis in neurons derived from iPSCs with 43 CAGs [89]. Adenosine receptor 2A agonists CGS-21680 and APEC reduced oxidative stress toxicity in the cells exposed to H_2_O_2_ by decreased *γ*-H2A Histone Family Member X (*γ*H2AX) induction and caspase 3 cleavage [90].

Other studies have shown that G-protein coupled receptor 52 (GPR52) is involved in mutant huntingtin toxicity. GPR52 knockdown as well as the microRNA 196a (miR196a) reduced huntingtin aggregates in neuronal culture [91]. Additionally, GPR52 knockdown lowered caspase 3 activity in response to BDNF withdrawal. Moreover, the number of condensed nuclei after BDNF withdrawal was reduced by peroxisome proliferator-activated receptor gamma (PPAR*δ*) activator (KD3010) [92]. Many research groups have described drug effect of BDNF or FGF/LIF withdrawal, considered as a stress factor on cell survival [88]. Inhibitor of ataxia-telangiectasia mutated (ATM) protein, a kinase involved in the DNA damage response, apoptosis, and cellular homeostasis, KU60019, applied on the 109 and 180 CAG mixed neuronal cell cultures has shown reduced BDNF withdrawal-induced increases in TUNEL-positive nuclei and reduced caspase 3 activity [93]. In 2015, Ring at al. demonstrated that TGF-*β* signaling pathway is altered in HD NSC with 72 CAGs that leads to higher expression of TGF-*β*. Furthermore, TGF-*β*1 plays neuroprotective role in the NSC models and together with netrin-1 can reduce caspase 3/7 activity [94].

Effects of compounds on PSCs phenotypes not related to a stressor addition have been tested by Charbord et al. The studies were associated with increased activity of the transcriptional repressor REST/NRSF binding to repressor element-1 (RE1) sequences in HD. High-throughput screening of thousands of selected compounds identified two benzoimidazole-5-carboxamide derivatives that inhibited REST silencing in a RE1-dependent manner. Treatment of 72 CAG NSCs with X5050 inhibitor, targeted REST degradation, increased the expressions of BDNF and other REST-regulated genes [95]. In 2013, Guo et al. developed a selective inhibitor (P110-TAT) of the mitochondrial fission protein dynamin-related protein 1 (DRP1). Treatment of iPSC-derived neurons from HD patients with P110-TAT reduced mitochondrial fragmentation and improved mitochondrial function. Furthermore, P110-TAT increased cell viability and reduced the extent of neurite shortening in neuronal culture [96].

## 4. Concluding Remarks

The iPSC technology is a novel and complementary approach to studying neurological diseases.

Generation of human iPSC-derived neuronal cells enables the understanding of neural development and neuropathological processes in a more accessible manner than has previously been achieved. Rapid progress is being made in improving techniques for differentiation of iPSCs towards diverse populations of neural subtypes. Generation of hiPSC-derived cholinergic neurons will broaden our knowledge about mechanisms of cholinergic function during development and synaptic plasticity. The ability to selectively control neuronal differentiation of PSCs into functional neurons expressing cholinergic neurotransmitters and receptors is a significant step in understanding neurodegenerative diseases. Furthermore, it can be used for the development of cell replacement therapy for Alzheimer's disease and high-throughput screening for agents that promote BFCNs survival. The in vitro generated biologically functional DA neurons offer a renewable source for toxicological and pharmaceutical drug screening. They may provide an effective tool in the development of sustainable therapies for disorders that affect the DA system such as Parkinson's disease.

Despite the above-mentioned advantages, there are still some hurdles in expanding the use of iPSCs to study neurological diseases. First, there is lack of uniformity in culture techniques and induction methods that make unclear how reproducible differentiation protocols are. Methods based on neuronal differentiation in three-dimensional systems seem to be more efficient than adherent two-dimensional cultures. However, it is not determined which system is more adequate for in vivo environment. Second, a better characterization of a given neural subtypes is needed as well as verification whether newly generated neurons are able to integrate into the circuitry of a host organism. Neurons derived from hiPSCs required few weeks of culture to become electrophysiologically active and few months to integrate into the host neural environment after transplantation. Thus, the proper model system must be considered for assessing the function of human stem cell derived neurons and evaluating their safety in the long-term culture. Another difficulty is related to a variability of the iPSCs phenotypes within the cell lines, even if they are derived from the same patient. The iPSC variations can originate from different sources during iPSC generation and maintenance. Therefore, strong emphasis should be placed on demonstrating that phenotype of iPSC-derived neurons is associated with specific genetic background and not with this variability. The potential source of the line-to-line variation is the reprogramming process that may disturb the genomic and epigenetic stability and potentially introduces de novo epigenetic variations. Furthermore, during a clonal selection process, clones carrying genetic defects can be selected, while the increasing passage number might introduce genetic alterations that facilitate cell propagation. Such genetic and epigenetic variations between different iPSCs lines may affect the differentiation potential and undermine the iPSCs accountability in downstream applications. Thus, several iPSC lines from the same individual are necessary to monitor the phenotypic variability between these lines.

Despite the above limitations, patient-specific iPSC-derived neurons provide an opportunity to model disease process in vitro. iPSCs carrying the genetic background and epigenetic pattern of the host individual can be a good tool to study the multifactorial diseases. One of the most challenging issues in disease modeling is the identification of the disease phenotype of neurons from patients. The difficulties are particularly visible in studying neurodegenerative disorders which evolve over several decades in vivo. Therefore, a reconstruction of cellular environment and interactions between the cells under the disease conditions can facilitate a proper phenotype development.

In conclusion, recent studies confirmed that iPSCs can accelerate drug discovery and might be applied for cell transplantation. In particular, ESC/iPSC-derived cholinergic and dopaminergic interneurons resulted in improved neurodegenerative diseases models, thus complementing animal models and accelerating the translation towards patient subgroup stratified clinical trials. We predict that iPSCs derived neural cells will be suitable to be used in various applications of in vitro disease modeling, high-throughput or high-content drug, and toxicology screening and potentially, in cell-transplant-mediated therapeutic interventions.

## Figures and Tables

**Table 1:** Neurological disorders modeled with patient-specific human induced pluripotent stem cells.

Disease	Genetic background	Disease related phenotype	Affected neurons	iPSCs model	References
Neurodegenerative disorders					

Alzheimer's disease	PS1, PS2, APP duplication, ApoE	(i) Increased A*β*42 secretion; (ii) A*β* plaque formation; (iii) increased phospho-Tau; (iv) increased GSK3*β* activity	Basal forebrain cholinergic neurons; cortical neurons	(i) AD iPSCs with E693 deletion in APP gene; (ii) AD iPSCs with mutation in APP (V717I); (iii) AD iPSCs with a duplication APP gene; (iv) AD iPSCs with PS1 (A246E) and PS2 (N141I) mutation	[40–43]

Parkinson's disease	SNCA, LRRK2, PARKN, PINK1, UCHL1, GBA	(i) *α*-Synuclein accumulation; (ii) reduced numbers of neuritis; (iii) increased susceptibility to oxidative stress; (iv) accumulation of ER-associated degradation substrates	Dopaminergic neurons	(i) PD iPSCs with triplication of the SNCA; (ii) PD iPSCs with *α*-synuclein mutation (A53T); (iii) PD iPSCs with G2019S mutation in LRRK2 gene; (iv) PD iPSCs with mutation in PINK1	[53, 54, 57, 63]

SMA	SMN1, SMN2	(i) Reduced SMN gene expression; (ii) Fas ligand-mediated apoptosis of MN; (iii) increased level of caspase 3, caspase 8, and membrane-bound Fas ligand; (iv) reduced size, axonal elongation, and neuromuscular junction production	Motor neurons	(i) iPSCs with SMN1 mutation from SMA type I patients	[64, 65]

ALS	SOD1, TDP-43, FUS, VAPB	(i) Neurofilament-L aggregation in neuritis; (ii) axonal degeneration; (iii) increased secretion of TDP-43; (iv) exhibited shortened neurites	Motor neurons	(i) ALS iPSCs with A4V SOD1 mutation; (ii) ALS iPSCs with D90A SOD1 mutation; (iii) ALS iPSCs with mutation invTDP-43 gene; (iv) ALS iPSCs with VAPB (P56S) mutation	[66–68]

Huntington's disease	HTT (CAG repeats)	(i) Increased vulnerability to cell stressors and BDNF withdrawal; (ii) impaired lysosomal activity; (iii) mitochondrial fragmentation; (iv) alterations in transcription repressor activity; (v) enhanced caspase 3/7 activity	Cortical neurons; GABAergic medium spiny neurons	(i) iPSCs with HTT mutation from homozygous and heterozygous HD patients HD72-iPSC in the YAC128 model of HD	[5, 69–72]

Neurodevelopmental disorders					

Familial Dysautonomia	IKBKAP	(i) Reduced IKAP protein level; (ii) cell migration deficiency; (iii) defects in neurogenic differentiation; (iv) decreased in number of myelinated small fibers and intermediolateral column neurons	Sensory neurons; autonomic neurons	(i) FD iPSCs with mutation in IKBKAP gene	[73]

Rett syndrome	MECP2e1, MECP2e2	(i) Reduced soma size; (ii) altered dendritic spine density; (iii) dysfunction in action potential; (iv) alterations in synaptic function; (v) defects in synaptic plasticity	Glutamatergic neurons	(i) RS iPSCs with MECP2 mutation	[74, 75]

ASD	NLGN1, NLGN3, SHANK2, SHANK3, NRXN1, NRXN3	(i) Reduced glial differentiation; (ii) altered gene expression related to cell adhesion and neuron differentiation; (iii) deficits in neuronal specification, synapse formation and excitatory neurotransmission	Cortical neurons	(i) ASD iPSCs with functional knockdown of NRXN1 gene; (ii) ASD iPSCs with SHANK3 deletion	[76, 77]

Down syndrome	trisomy of chromosome 21 (HSA21)	(i) Alterations in neurogenesis and synaptogenesis; (ii) elevated APP and A*β*42 expression; (iii) Tau protein hyperphosphorylation; (iv) poorly developed neural network; (v) overproduction of reactive oxygen species	Neurons in the brain	(i) DS iPSCs with three pairs of chromosomes 21 (T21-iPSCs); (ii) isogenic iPSCs from DS individuals; (iii) DS iPSCs with trisomy 21 deletion through TKNEO; (iv) DS iPSCs with trisomy 21 deletion through Xist	[70, 78–80]

Schizophrenia	DISC1	(i) Decreased neuronal connectivity; (ii) synaptic deficits; (iii) PSD95 downregulation; (iv) fewer neurites	Neurons	(i) iPSCs from schizophrenia patients; (ii) SZ iPSCs with a mutation in DISC1 gene	[81, 82]

## Abbreviations

2D: 2-dimensional
3D: 3-dimensional
3-NT: 3-Nitrotyrosine
6-OHDA: 6-Hydroxydopamine
AA: Ascorbic acid
A*β*: Amyloid beta
ACh: Acetylcholine
AChE: Acetylcholinesterase
AD: Alzheimer's disease
ALS: Amyotrophic lateral sclerosis
APP: Amyloid precursor protein
APP-CTF: C-terminal fragments of amyloid precursor protein
ASD: Autism spectrum disorder
ATM: Ataxia-telangiectasia mutated
BDNF: Brain derived neurotrophic factor
BFCN: Basal forebrain cholinergic neurons
BMP: Bone morphogenic protein
BMP9: Bone morphogenic protein 9
cAMP: Cyclic adenosine 3′,5′-monophosphate
ChAT: Choline acetyltransferase
CDX2: Caudal type homeobox 2
CNS: Central nervous system
CNTF: Ciliary neurotrophic factor
DA: Dopaminergic
DAPT: N-[N-(3,5-Difluorophenacetyl)-1-alanyl]-S-phenylglycine t-butyl ester
DAT: Dopamine transporter
DHA: Docosahexaenoic acid
DNM1L: Dynamin 1-like
DRP1: Dynamin-related protein 1
EGF: Epidermal growth factor
EN1: Engrailed 1
ER: Endoplasmic reticulum
fAD: Familial Alzheimer's disease
FGF: Fibroblast growth factor
FGF8: Fibroblast growth factor 8
FOXA2: Fork head box protein A2
FP: Floor plate
GBA: Beta-glucocerebrosidase
GBX1: Gastrulation brain homeobox 1
GDNF: Glial cell line derived neurotrophic factor
GPR52: G-protein coupled receptor 52
GSK3B: Glycogen synthase kinase 3 beta
GSK3Bi: GSK3B inhibitor
GSMs: *γ*-Secretase modulators
GSAP: *γ*-Secretase activating protein
HD: Huntington's disease
hESC: Human embryonic stem cell
hiPSC: Human induced pluripotent stem cell
hPSC: Human pluripotent stem cell
HTT: Huntingtin
ISL1: ISL LIM homeobox 1
KLF4: Kruppel-like factor 4
KSR medium: Knockout serum replacement
Lhx6: LIM homeobox 6
Lhx8: LIM homeobox 8
LM511-E8: Recombinant E8 fragments of human laminin 511
LMX1A: LIM homeobox transcription factor 1 alpha
LRRK2: Leucine-rich repeat kinase 2
LTP: Long-term potentiation
mESC: Mouse embryonic stem cell
MGE: Median ganglionic eminence
miR196a: microRNA 196a
NCAM: Neural cell adhesion molecule
NES: Nestin
NFT: Neurofibrillary tangle
NGF: Nerve growth factor
NGFR: Nerve growth factor receptor
NKX2-1: NK2 homeobox 1
NRSF: Neuron restrictive silencer factor
NSAIDs: Nonsteroidal anti-inflammatory drugs
NSC: Neural stem cell
NTF3: Neurothrophin 3
NTF4: Neurothrophin 4
NTN1: Netrin 1
OCT4: Octamer-binding transcription factor 4
OTX2: Orthodenticle homeobox 2
PARK2: Parkin RBR E3 Ubiquitin Protein Ligase
PAX6: Paired box 6
PD: Parkinson's disease
PDL: Poly-D-Lysine
PKC: Protein kinase C
PPAR*δ*: Peroxisome proliferator activated receptor gamma
PPARGC1A: Peroxisome proliferator-activated receptor gamma, coactivator 1 alpha
PINK1: PTEN-induced putative kinase 1
PNS: Peripheral nervous system
POU5F1: POU domain, class 5, transcription factor 1
PSEN1: Presenilin 1
PSEN2: Presenilin 2
RA: Retinoic acid
RE1: Repressor element-1
REST: Repressor element-1 silencing transcription factor
ROCK: Rho-associated coiled-coil forming protein serine/threonine kinase
sAD: Sporadic Alzheimer's disease
SHH: Sonic hedgehog
SMA: Spinal muscular atrophy
SNC: Substantia nigra pars compacta
SNCA: Synuclein alpha
SOX1: Sex determining region Y-box 1
SOX2: Sex determining region Y-box 2
TGFB3: Transforming growth factor, beta 3
TH: Tyrosine hydroxylase
TNF*α*: Tumor necrosis factor-alpha
TUBB3: Tubulin, Beta 3 Class III
UCHL1: Ubiquitin carboxyl-terminal esterase L1
VAChT: Vesicular acetylcholine transporter
WNT: Wingless-Type MMTV Integration Site Family.
